# Supplementary material for: EVOO Polyphenols Relieve Synergistically Autophagy Dysregulation in a Cellular Model of Alzheimer’s Disease
Source: Int J Mol Sci. 2021 Jul 5;22(13):7225. doi: 10.3390/ijms22137225 (PMC8267626; doi:10.3390/ijms22137225)
Supplement: Supplementary file 1 [file ijms-22-07225-s001.zip › ijms-1223029-supplementary.pdf]

Supplementary Materials

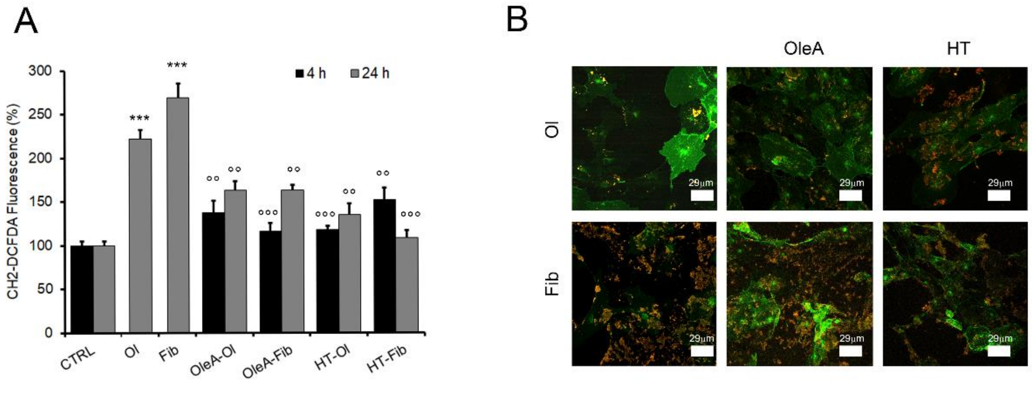

Supplementary Figure S1. Polyphenols effects on $A\beta_{1-42}$ treated cells. ROS production in SH-SY5Y cells treated for 24 h with 2.5 μM $A\beta_{1-42}$ oligomers (Ol) or fibrils (Fib) in absence or after cell pre-treatment with 75 μM OleA or HT for 4 h (black bars), 24 h (grey bars) (OleA-Ol, HT-Ol) or fibrils (OleA-Fib, HT-Fib) assessed by (**A**) CH2-DCFDA probe. DCFDA fluorescence is reported as percentage respect to untreated control cells. Error bars indicate the standard error of three independent experiments carried out in triplicate. ***: p-value <0.001 vs untreated control cells. °°: $p < 0.01$; °°° $p < 0.001$ vs $A\beta_{1-42}$ aggregates-treated cells. (**B**) Confocal microscopy showing the co-immunolocalization of $A\beta_{1-42}$ aggregates (Ol, Fib) and the membrane GM1 ganglioside in SH-SY5Y cells in the absence (Ol, Fib) and in the presence of pre-treatment with OleA or HT for 24 h. The cells were stained with Alexa 488-conjugated CTX-B probe (green staining); $A\beta_{1-42}$ aggregates were stained with anti-$A\beta_{1-42}$ primary antibody and then with Alexa 568-conjugated anti-rabbit secondary antibody (red fluorescence).

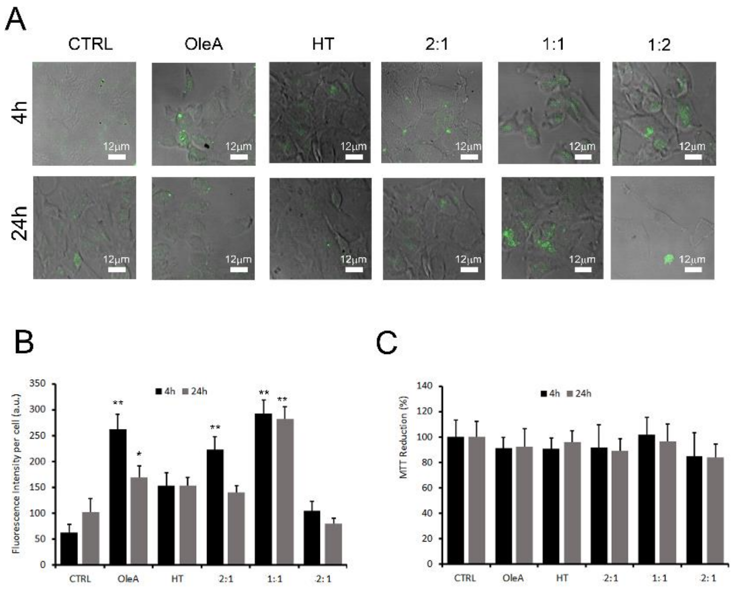

Supplementary Figure S2. Autophagy detection in SH-SY5Y cells exposed to polyphenols alone or mixed (MIX). (**A**) SH-SY5Y cells were treated with 75μM OleA or HT either separately or in mixture at different OleA/HT molar ratios (1:1, 37.5μM:37.5μM; 1:2, 25μM:50μM;2:1, 50μM:25μM), for two lengths of time (4 h and 24 h). The autophagosomes (green) were labelled with the Cyto-ID® fluorescent dye. (**B**) Quantification of green fluorescence per cell obtained from three independent experiments and three different fields (about 20 cells/field). * $p < 0.05$; ** $p < 0.01$ vs untreated cells (CTRL). (**C**) MTT assay of SH-SY5Y exposed at the different conditions. Error bars indicate the standard error of three independent experiments carried out in triplicate.
